# Managing Trait Trade-Offs in Tomato Breeding Through Genome Editing

**DOI:** 10.3390/plants15192994

**Published:** 2026-09-30

**Authors:** Jia Chae, Seong Ju Han, Hye Jeong Kim, Jee Hye Kim, Hyeon Ju Nam, Young-Soo Chung, Sivabalan Karthik, Jae Bok Heo

**Affiliations:** 1 Department of Molecular Genetic Engineering, Dong-A University, Busan 49315, Republic of Korea; cowldk14@gmail.com (J.C.); hsj9587@gmail.com (S.J.H.); hjkim83@dau.ac.kr (H.J.K.); kjh48524852@gmail.com (J.H.K.); hyunju030617@naver.com (H.J.N.); chungys@dau.ac.kr (Y.-S.C.); 2 Department of Microbiology, Thiagarajar College, Madurai 625009, Tamil Nadu, India; 3 National Center of Excellence in Statistical and Mathematical Modelling on Bio-Resources Management, Thiagarajar College, Madurai 625009, Tamil Nadu, India

**Keywords:** tomato, CRISPR-Cas, trait trade-offs, cis-regulatory editing, allele engineering, precision breeding

## Abstract

Tomato improvement often involves trade-offs between fruit yield and quality, sweetness and fruit size, disease resistance and growth, reproductive performance and stress adaptation, and shelf life and flavour. Although CRISPR-Cas-mediated genome editing enables targeted modification of tomato genes, DNA-level precision does not guarantee an agronomically favourable phenotype because pleiotropy, functional redundancy, allele dosage, linkage, epistasis, genetic background, and environmental conditions can alter editing outcomes. This review evaluates genome editing as a strategy to manage, rather than merely document, these trade-offs. We examine how knockout, multiplex, cis-regulatory, base, prime, and direct allele-repair approaches can match the causal architecture of a breeding constraint. We critically assess representative studies involving plant architecture, reproductive development, fruit size, sugar accumulation, ripening, nutritional composition, disease resistance, and heat resilience, with an emphasis on heritability, molecular identity, multi-trait effects, and agronomic penalties. We further consider domestication-associated variation, structural variation, transformation and regeneration limitations, genotype-by-environment interactions, breeding deployment, and regulatory translation. We propose a trade-off-aware framework that links causal-allele identification, mechanism-matched editing, quantitative allele design, multidimensional phenotyping, and validation across generations, genetic backgrounds, and production-relevant environments. This framework can guide the development of balanced, breeding-ready tomato cultivars rather than lines optimized for a single trait.

## 1. Introduction

Tomato (*Solanum lycopersicum* L.) is an economically important horticultural crop and a widely used experimental system for studying fleshy-fruit development, ripening, metabolism, and domestication. Publication of the tomato reference genome provided a reference sequence for identifying genes and genomic regions associated with agronomic and fruit-quality traits [1]. Population-genomic analysis subsequently showed that domestication and modern improvement imposed distinct selection pressures on loci associated with fruit size and other breeding traits [2]. Flavour-related characteristics, however, were not consistently retained during this process. A meta-analysis of genome-wide association studies involving 775 tomato accessions showed that domestication and improvement altered the allelic composition of loci associated with sugars, organic acids, amino acids, and flavour-related volatile compounds [3].

The genetic variation available for tomato improvement extends well beyond the variation represented by a single reference genome. A tomato pangenome constructed from 725 accessions identified genes absent from the reference genome and recovered a rare allele of *TomLoxC* associated with fruit flavour [4]. A subsequent graph-based pangenome constructed from 838 tomato genomes catalogued more than 19 million variants and increased the proportion of variation explained for complex molecular and agronomic traits, including soluble-solids content [5]. These resources have expanded the range of candidate genes and alleles available for breeding. Nevertheless, identification of a favourable locus does not establish that modifying it will improve one characteristic without affecting other agronomically important traits.

An early application of CRISPR-Cas9 in tomato demonstrated efficient and heritable mutagenesis of *ARGONAUTE7*, including recovery of edited plants in the first transformed generation [6]. Genome editing was subsequently used to modify six domestication-associated loci in wild *Solanum pimpinellifolium*, producing plants with altered architecture and larger and more numerous fruits while maintaining the high lycopene accumulation associated with the wild material [7]. These studies confirmed that targeted editing could support both gene-function analysis and the modification of domestication traits in tomato. However, precision at the DNA-sequence level does not necessarily produce an equally restricted phenotypic effect. Complete loss-of-function alleles may alter several traits when the target gene is pleiotropic. In contrast, disruption of one gene may produce a weak or undetectable phenotype when related genes provide functional compensation.

In this review, a trait trade-off refers to an improvement in one breeding target that is accompanied by an undesirable or context-dependent change in another agronomically important trait. We distinguish five principal genetic mechanisms: direct pleiotropy, allele-dosage effects, epistasis, linkage or structural variation, and functional redundancy. Representative tomato studies have shown that allele strength, pleiotropy, epistasis, functional redundancy, and reproductive effects can determine whether an edit produces an agronomically balanced phenotype [8,9,10,11,12]. Studies involving precise allele repair and alternative delivery approaches further demonstrate that breeding value depends on identifying the causal sequence, recovering heritable edits, and regenerating suitable plants [13,14,15]. Genetic background and environmental conditions are therefore treated as modifiers of the five mechanisms because they can alter the direction or magnitude of an edited phenotype. An editing strategy should therefore be selected based on the causal architecture of the breeding constraint rather than editing efficiency alone.

Accordingly, this review has three objectives: (i) to determine how direct pleiotropy, allele dosage, epistasis, linkage or structural variation, and functional redundancy generate or expose trait trade-offs in tomato breeding; (ii) to evaluate how loss-of-function, multiplex, cis-regulatory, base-editing, prime-editing, and allele-repair strategies can be matched to these mechanisms; and (iii) to define the molecular, phenotypic, genetic, and environmental evidence required to demonstrate mitigation rather than redistribution of a trade-off. The discussion therefore proceeds from mechanistic diagnosis and selection of an appropriate editing strategy to evaluation of representative tomato applications, experimental validation, and breeding and regulatory deployment.

## 2. Genetic Mechanisms Underlying Trait Trade-Offs in Tomato

Managing a trait trade-off requires identification of the genetic relationship between the intended breeding target and the competing phenotype. Tomato domestication and improvement selected alleles affecting fruit size, plant architecture, harvesting characteristics, and fruit composition [2,3], while pangenome and graph-pangenome analyses revealed gene-content, sequence, and structural variation that is not represented by a single reference genome [4,5]. The outcome of an edit may therefore depend on direct pleiotropy, allele dosage, epistasis, physical linkage or structural variation, functional redundancy, and the genetic background in which the edit is introduced. These mechanisms provide the basis for selecting an appropriate editing response.

### 2.1. Pleiotropy and Developmental Coupling

Pleiotropy occurs when a single gene contributes to more than one phenotypic process. It is an important source of breeding trade-offs because genes controlling meristem activity, flowering, inflorescence architecture, fruit development, ripening, and resource allocation can influence several agronomic characteristics. Complete disruption of a pleiotropic gene may improve the intended trait while impairing another function that is important for productivity, reproduction, or fruit quality.

CRISPR-Cas9 editing of tomato promoters demonstrated that cis-regulatory mutations can generate quantitative allelic series for fruit size, inflorescence branching, and plant architecture [8]. Different promoter alleles produced phenotypic changes of different magnitudes, showing that the outcome depends not only on the targeted gene but also on the regulatory sequence and site being modified [8]. This approach helps identify intermediate alleles when complete loss of function produces an excessively strong or agronomically undesirable phenotype.

Cis-regulatory dissection of *WOX9* provided direct evidence of previously unresolved pleiotropy. A comprehensive series of regulatory alleles revealed multiple roles for *WOX9* in reproductive development, while analyses of accessible chromatin and conserved non-coding sequences identified regulatory regions contributing to these effects [9]. Functional comparisons involving groundcherry and *Arabidopsis thaliana* further indicated evolutionary conservation of aspects of *WOX9* regulatory activity [9]. These findings support the use of cis-regulatory editing to dissect pleiotropic functions and identify alleles with more restricted phenotypic effects. Nevertheless, regulatory editing does not inherently eliminate trade-offs because the resulting alleles can still influence multiple developmental processes.

Editing *SlCDPK27* and its paralogue *SlCDPK26* provides another instructive example. Loss-of-function alleles increased fruit glucose and fructose contents by up to approximately 30% without detectable penalties in fruit weight or total yield under the reported experimental conditions [12]. However, the edited plants produced fewer and lighter seeds, although germination was not impaired [12]. Thus, the principal relationship between increased sugar content and reduced fruit size or yield was substantially uncoupled, but an additional reproductive phenotype remained. Claims that an edit has eliminated a trade-off should therefore identify the traits, genotypes, developmental stages, and environmental conditions that were evaluated.

### 2.2. Epistasis, Cryptic Variation, and Allele Dosage

Epistasis occurs when the phenotypic effect of one allele depends on the state of another locus. This is particularly important in tomato because domestication and modern breeding have assembled alleles originating from different populations, wild relatives, and breeding programmes. Consequently, an allele that is favourable in one genetic background may be neutral or deleterious in another. Natural and engineered cis-regulatory alleles in a tomato stem-cell regulatory circuit produced idiosyncratic and dosage-dependent interactions affecting fruit size [10]. The phenotypes of combined alleles could not always be predicted by adding the effects of the corresponding individual alleles [10]. Quantitative edits should therefore be evaluated in the allele combinations relevant to the recipient cultivar, especially when several components of the same developmental pathway are being modified.

The interaction between *JOINTLESS2* (*J2*) and *ENHANCER OF JOINTLESS2* (*EJ2*) provides a well-characterized example of negative epistasis. Tomato domestication selected for a weak *ej2* allele. In contrast, breeders later used a mutation in its MADS-box paralogue *J2* to eliminate the fruit-stem abscission zone and facilitate harvesting. Combining these alleles produced excessive inflorescence branching, reduced fertility, and lower productivity [16]. Analysis of natural and CRISPR-generated alleles showed that graded changes in MADS-box gene activity generated a range of inflorescence architectures and that suitable allele combinations could improve productivity while retaining useful harvesting characteristics [16].

A subsequent study showed that a tandem duplication carrying the partially mis-spliced *ej2* allele increased the abundance of functional transcripts above the threshold required to suppress excessive branching [17]. The duplication restored more normal flowering and fertility in the presence of the jointless breeding allele [17]. Dissection of this dosage mechanism also enabled more predictable CRISPR-Cas9 strategies for introducing the jointless harvesting phenotype [17]. Together, these studies show that a cryptic allele with little effect by itself can produce a major breeding penalty when combined with another mutation and that copy-number variation can modify the strength of this interaction [16,17].

### 2.3. Linkage, Structural Variation, and Selection-Associated Metabolic Changes

Not all trade-offs result from a single pleiotropic gene. A favourable allele can be inherited with neighbouring undesirable variants because of physical linkage. This problem is particularly relevant when chromosome segments from wild relatives are introduced to obtain disease resistance, abiotic-stress tolerance, or fruit-quality characteristics. When recombination does not separate the desired allele from neighbouring functional variants, selection for one trait can alter other phenotypes. Flavour deterioration in modern tomato is an important example of selection-associated change. Chemical and genomic analyses of modern, heirloom, and wild tomato accessions identified compounds contributing to consumer preference. They showed that favourable alleles associated with several flavour-related chemicals became less frequent during breeding [18]. Because tomato flavour depends on combinations of sugars, organic acids, and volatile compounds, its deterioration cannot be attributed to one universal “flavour gene” [3,18].

Integrated analysis of tomato genomes, transcriptomes, and metabolomes demonstrated that selection for larger fruits altered metabolite profiles partly because fruit-weight loci were linked to nearby genes affecting metabolism [19]. Selection at five major loci contributed to reduced accumulation of antinutritional steroidal glycoalkaloids in ripe fruit, whereas breeding for pink fruit affected the abundance of more than 100 metabolites [19]. Introgression of disease-resistance genes from wild relatives was also associated with substantial, sometimes unexpected, metabolic changes [19]. These observations help distinguish linkage-associated effects from direct pleiotropy: a metabolic phenotype may change because functional variants are inherited together rather than because one gene directly controls both traits.

Structural variation further complicates these relationships. Long-read sequencing of 100 diverse tomato accessions identified 238,490 structural variants, including variants intersecting genes and cis-regulatory regions [20]. Numerous structural variant-gene pairs were associated with differences in gene expression, and experimental analyses showed that structural variants affecting gene dosage or expression contributed to variation in fruit flavour, size, production, and harvesting traits [20]. The relevant functional unit can therefore be a promoter rearrangement, duplication, deletion, or multi-gene haplotype rather than a single-nucleotide polymorphism.

Direct editing of a validated causal allele could reduce linkage drag by avoiding the transfer of an extended donor chromosome segment into an elite cultivar. However, this advantage depends on correctly distinguishing the causal variant from neighbouring markers. Editing a candidate gene without accounting for surrounding haplotype and structural variation may fail to reproduce the donor phenotype. Target validation should therefore integrate reference-genome annotation with accession-specific pangenome, graph-pangenome, and structural-variation information [4,5,20].

### 2.4. Functional Redundancy and Compensatory Networks

Gene duplication can generate paralogues with overlapping, partially overlapping, or divergent functions. When paralogues compensate for one another, disruption of a single gene may produce a weak or undetectable phenotype. Conversely, simultaneous disruption of several family members may remove biological buffering and produce a stronger or more pleiotropic phenotype than anticipated.

Researchers developed a genome-scale multi-target CRISPR resource in tomato to investigate such functional redundancy. The library contained 15,804 guide RNAs targeting multiple members of the same gene families and was used to generate approximately 1300 independent edited lines [11]. The screen recovered mutants with phenotypes related to fruit development, flavour, nutrient acquisition, and pathogen responses [11]. This work demonstrated that multiplex editing can reveal functions masked by redundancy. It also emphasized the need to calibrate the number and combination of paralogues disrupted because complete removal of compensatory activity may expose additional developmental or physiological effects.

Comparative pan-genetic analysis across *Solanum* species showed that the outcome of editing an evolutionarily conserved target can depend on the presence and functional state of related genes [21]. Lineage-specific duplication, loss, pseudogenization, and functional diversification of paralogues changed the genetic context in which developmental regulators operated [21]. Accordingly, conservation of the target-gene sequence alone is insufficient evidence that an editing strategy will produce equivalent phenotypes across cultivars or related species.

Hierarchical epistasis among paralogous gene pairs adds another level of complexity. Analysis of the tomato *PLT3/PLT7–J2/EJ2* regulatory network showed that combinations of mutations could expose cryptic effects, generate synergistic phenotypes within paralogue pairs, or mask the effects of additional mutations elsewhere in the network [22]. As the phenotypic effect of perturbing one paralogue pair increased, the contribution of mutations in the other pair progressively diminished [22]. Functional redundancy should therefore not be interpreted simply as two interchangeable genes performing the same function. Paralogues can form dosage-sensitive networks in which editing one member depends on the functional states of several others.

### 2.5. Genetic Background as a Cross-Cutting Variable

The *J2/EJ2* examples demonstrate that a pre-existing domestication allele can convert a useful harvesting mutation into an inflorescence-branching and fertility penalty [16,17]. Quantitative fruit-size alleles similarly show that phenotypic effects can depend on allele dosage and gene combination [10]. Structural-variation and pan-genetic analyses further establish that gene copy number, regulatory variation, and paralogue composition differ among tomato accessions and related *Solanum* species [20,21,22].

Genetic background should therefore be treated as an explicit component of target selection and experimental design. Before editing, the recipient cultivar should be examined for the target-gene sequence, copy number, known structural variants, functional paralogues, and established interacting alleles. When the target belongs to a developmental or metabolic network, relevant linked and epistatic loci should also be characterized. Where feasible, the same edit should be evaluated in more than one agronomically relevant background before generalizing its effects across tomato germplasm.

### 2.6. Mechanistic Classification and Editing Implications

For genome-editing applications, tomato trait trade-offs fall into five mechanistic categories. First, direct pleiotropic trade-offs occur when the target gene affects multiple biological processes. Second, dosage-dependent trade-offs arise when complete loss of function produces a greater change than is required for the desired phenotype. Third, epistatic trade-offs occur when the edited allele interacts non-additively with another domestication, improvement, or cryptic allele. Fourth, linkage-associated trade-offs result from neighbouring variants or extended introgressed haplotypes rather than from the intended target itself. Fifth, redundancy-associated trade-offs arise when paralogues either compensate for a single edit or generate excessive phenotypic effects following multiplex disruption.

**Figure 1:**
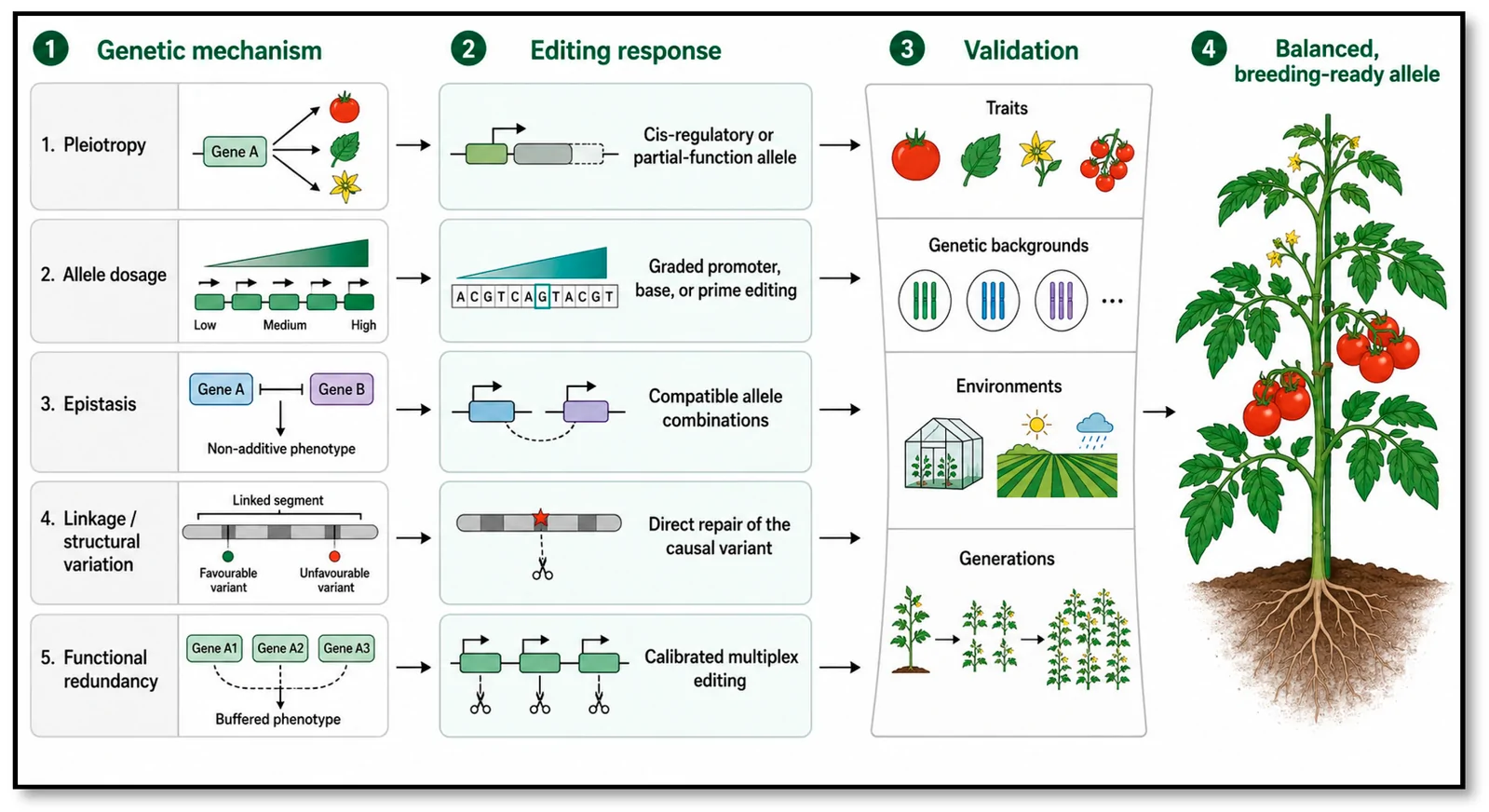
Trade-off-aware framework for selecting genome-editing strategies in tomato. Promoter editing is a form of cis-regulatory editing that can generate quantitative allelic variation by modifying gene-expression level, timing, or tissue specificity. Pleiotropy, dosage sensitivity, epistasis, linkage or structural variation, and functional redundancy require mechanism-matched editing responses. Candidate alleles must subsequently undergo multi-trait validation across genetic backgrounds, environments, and generations before breeding deployment. Solid arrows indicate the direction of mechanistic effects or progression through the framework; blunt-ended lines indicate non-additive interactions; and dashed lines indicate functional or combinatorial relationships. The red star marks the causal variant selected for direct repair, and the scissors indicate editing sites. The ellipsis under “Genetic backgrounds” denotes additional recipient backgrounds beyond the three illustrated.

Diagnosis of the underlying mechanism narrows the range of suitable editing responses. Pleiotropic or dosage-sensitive targets generally require quantitative or condition-dependent changes in activity rather than complete loss of function. Epistatic trade-offs require evaluation of compatible allele combinations, whereas direct editing at linkage-associated or structurally complex loci is appropriate only after resolving the causal variant and local haplotype. Functional redundancy may require multiplex editing, but the number and combination of paralogues disrupted must be calibrated to avoid excessive phenotypic effects. Figure 1 outlines the relationship among causal mechanism, editing response, and breeding-level validation.

## 3. Matching Genome-Editing Strategies to Trade-Off Mechanisms

Once the mechanism underlying a trade-off has been identified, the editing strategy should be selected according to the required phenotypic change and the biological functions that must be preserved. Loss-of-function editing is appropriate when strong suppression of a nonessential negative regulator or susceptibility factor is required and biologically tolerable. Multiplex editing may be necessary when functional redundancy or interactions among loci limit the effect of a single mutation. Cis-regulatory editing is better suited to pleiotropic or dosage-sensitive genes whose expression level, developmental timing, or tissue specificity must be adjusted without eliminating protein function. Base editing, prime editing, and direct allele repair are most relevant when a defined sequence change can reproduce or restore a favourable allele. Strategy selection should therefore be guided by the causal mechanism, required allelic effect, and recipient genetic background rather than editing efficiency alone.

### 3.1. Loss-of-Function Editing for Biologically Tolerable Targets

Loss-of-function editing is appropriate when eliminating a nonessential negative regulator or susceptibility factor is necessary and biologically tolerable. Disruption of the susceptibility factor *SlMlo1* conferred powdery mildew resistance without a detected reduction in harvested fruit weight under the reported conditions [23]. By contrast, simultaneous disruption of *SlCDPK27* and *SlCDPK26* increased fruit glucose and fructose concentrations without detectable reductions in fruit weight or total yield but reduced seed number and seed weight [12]. These examples show that knockout can either avoid an evident penalty among the traits measured or shift the biological cost to another component of performance. Its breeding value must therefore be assessed across the intended trait, vegetative development, reproduction, seed performance, yield, and relevant environmental conditions.

### 3.2. Multiplex Editing

Multiplex editing enables the simultaneous modification of multiple genes or regulatory regions. It is particularly useful for functionally redundant paralogues, multigene pathways, domestication syndromes, and traits requiring coordinated changes at several loci. A genome-scale tomato resource comprising 15,804 sgRNAs targeting multiple members of gene families generated approximately 1300 independent edited lines and identified phenotypes related to fruit development, flavour, nutrient acquisition, and pathogen responses [11]. This work demonstrated that multi-target editing can reveal phenotypes that remain concealed when only one family member is disrupted.

Multiplex editing has also enabled the reconstruction of domestication-associated traits in wild tomato backgrounds. Editing six domestication-associated loci in *Solanum pimpinellifolium* modified plant architecture and increased fruit size and number while retaining several characteristics of the wild genetic background [7]. However, the experiment did not demonstrate that every unmeasured component of wild adaptation remained unaffected. Its conclusions should therefore remain limited to the traits and conditions evaluated.

A complementary study introduced desirable traits into four stress-tolerant wild tomato accessions using multiplex CRISPR-Cas9 editing of coding sequences, cis-regulatory regions, and upstream open reading frames associated with plant morphology, flower and fruit production, and ascorbic acid synthesis [24]. Progeny were reported as Cas9-free, indicating that the assay did not detect the Cas9 sequence; they displayed domestication-related phenotypes while retaining the disease resistance or salt tolerance of their respective parental accessions [24]. This strategy illustrates how defined domestication traits can be introduced without replacing extended regions of a stress-adapted wild genome.

Multiplex editing nevertheless increases the possibility of non-additive genetic effects. Natural and engineered cis-regulatory alleles in the *SlCLV3–SlWUS* stem-cell regulatory pathway produced saturating, dosage-dependent, and allele-specific epistatic effects on locule number and fruit size [10]. Consequently, combining individually favourable edits does not guarantee an additive improvement. A strong allelic combination may exceed the optimal biological range, whereas another combination may provide little additional benefit because the pathway response has reached saturation.

Multiplex experiments should therefore include the corresponding single-edited genotypes, informative intermediate combinations, and the complete multiplex genotype. This design distinguishes the effects of functional redundancy, allele dosage, additivity, and epistasis. Whenever feasible, edits should be assembled progressively rather than combining the strongest available allele at every locus from the outset.

### 3.3. Cis-Regulatory Editing for Dosage-Sensitive and Pleiotropic Targets

Cis-regulatory editing is appropriate when complete loss of gene function would produce unacceptable pleiotropic or dosage-dependent effects. Promoter editing, a form of cis-regulatory editing, can generate quantitative allelic series that tune fruit size, inflorescence branching, and plant architecture, allowing selection of intermediate phenotypes rather than complete gene disruption [8]. However, regulatory alleles of *WOX9* and components of the *SlCLV3–SlWUS* pathway demonstrate that altered gene expression can retain pleiotropic, nonlinear, and genetic-background-dependent effects [9,10]. Candidate alleles should therefore be compared for target-gene expression in relevant tissues and developmental stages and for their effects on both intended and competing traits in the recipient genetic background. Cis-regulatory editing can adjust the strength or context of gene activity, but it does not automatically eliminate a trade-off.

### 3.4. Base Editing for Causal-Allele Repair

Base editing is appropriate when a validated nucleotide substitution can reproduce or repair a favourable allele while preserving the target gene’s broader function. Repair of the causal nucleotide in the domesticated *SSP2* allele produced compact plants and increased early fruit production without transferring an extended donor haplotype [13]. Tomato Target-AID studies established the recovery of heritable base substitutions [25] and showed that different substitution combinations in *SlDDB1*, *SlDET1*, and *SlCYC-B* produced distinct carotenoid profiles accompanied by dwarfing and other high-pigment-associated phenotypes [26]. Thus, nucleotide-level precision does not ensure phenotypic specificity, particularly when the edited genes regulate several developmental or metabolic processes. Base editing is most suitable when the causality of the variant is established and modifying a specific nucleotide is preferable to complete gene disruption; the resulting allele must still be evaluated for molecular identity, inheritance, and effects on competing agronomic traits.

### 3.5. Prime Editing for Defined Sequence Changes

Prime editing is appropriate when a validated favourable allele requires a substitution, short insertion, deletion, or multi-nucleotide replacement that cannot readily be generated by conventional base editing. Studies in tomato have established the recovery and inheritance of prime-edited alleles, while also showing that mosaicism, target dependence, and unintended local sequence products remain important constraints [27,28]. Its relevance to trade-off management was demonstrated by inserting a 10-bp heat-shock element into a cell-wall-invertase promoter, thereby producing heat-responsive gene activation and an approximately 33% increase in per-plot yield under heat stress without detected penalties in the fruit-quality traits measured in that study [29]. More recent optimized systems have broadened the range and combination of recoverable edits, although imprecise products remain target-dependent [30]. Therefore, prime-editing systems should be selected based on the required biological change rather than editing efficiency alone, and the resulting alleles should be evaluated for sequence identity, inheritance, and multi-trait performance under relevant genetic and environmental conditions.

### 3.6. Selecting the Appropriate Editing Modality

In general, the least disruptive edit capable of producing the required phenotypic improvement should be preferred. The appropriate strategy depends on the causal architecture of the trade-off, the required allelic strength, the recipient genetic background, and the biological functions that must be preserved.

A robust workflow should first establish whether the trade-off results from complete gene activity, gene dosage, functional redundancy, a defined causal nucleotide, physical linkage, or an interaction among loci. Knockout is appropriate when strong loss of function is both necessary and biologically tolerable. Cis-regulatory editing is preferable when the phenotype depends on expression level, developmental timing, tissue specificity, or environmental context. Base editing is suited to validated and chemically accessible nucleotide substitutions, whereas prime editing is appropriate for sequence changes outside conventional base-editing capabilities. When several loci contribute to the phenotype, multiplex edits should be introduced progressively.

## 4. Genome-Editing Applications to Major Tomato Trait Trade-Offs

The valuable breeding application of a genome-editing strategy depends on whether it improves the target trait without imposing an unacceptable cost on another agronomic characteristic. The examples examined in this section illustrate how mechanism-matched editing has alleviated, retained, or redistributed trade-offs involving fruit composition, productivity, disease resistance, reproductive performance, stress adaptation, and plant architecture. Each case is considered in relation to the underlying mechanism, the editing response, the competing traits evaluated, and the limitations of the available evidence.

### 4.1. Fruit Sweetness Versus Fruit Size, Yield, and Reproductive Performance

Fruit sugar concentration and fruit size are generally negatively associated during tomato domestication and improvement. Selection for large-fruited cultivars altered the frequency of alleles associated with soluble-solids content and other flavour-related characteristics, contributing to a persistent breeding conflict between consumer-preferred sweetness and commercial productivity [3,12]. However, this inverse association does not mean that increased sugar accumulation must invariably reduce fruit size or yield.

The identification of *SlCDPK27* and its paralogue *SlCDPK26* provided a mechanistically defined route to modify this relationship. The encoded calcium-dependent protein kinases phosphorylate the sucrose synthase *SlSUS3*, promoting its degradation and thereby restricting sugar accumulation during fruit ripening [12]. Simultaneous genome editing of *SlCDPK27* and *SlCDPK26* increased glucose and fructose concentrations by up to approximately 30% without detectable reductions in individual fruit weight or total yield under the experimental conditions examined [12]. Consumer-panel evaluation also indicated increased perceived sweetness of the edited fruit [12].

Nevertheless, the improvement was not entirely cost-free. Double-knockout plants produced fewer and lighter seeds, although seed germination was not impaired [12]. The principal fruit size–sweetness relationship was therefore substantially alleviated, but part of the biological cost was redistributed to seed production. This distinction is important because reproductive performance has different commercial implications in hybrid-seed production, processing tomatoes, fresh-market cultivars, and experimental germplasm.

This example also demonstrates that yield neutrality must be defined using multiple components. Comparable individual fruit weight and total yield do not exclude changes in fruit number, seed number, seed weight, flowering, or performance under environmental stress. Edited high-sugar genotypes should therefore be evaluated for soluble solids, individual sugars, organic acids, fruit weight, fruit number, total yield, seed characteristics, germination, and sensory quality.

Fruit size is itself controlled by nonlinear interactions among developmental regulators. Natural and engineered cis-regulatory alleles in the *SlCLV3–SlWUS* stem-cell pathway produced saturating, dosage-dependent, and allele-specific epistatic effects on locule number and fruit size [10]. Consequently, combining a sweetness-enhancing mutation with a fruit-size allele cannot be assumed to produce independent or additive effects. The strength of each allele and the genetic background in which they are combined must be considered when reconstructing the fruit size–sweetness relationship.

### 4.2. Nutritional Enrichment Versus Plant Growth and Metabolic Balance

Genome editing can increase nutritionally relevant metabolites by redirecting metabolic flux or modifying enzyme regulation. However, primary and specialized metabolic pathways are interconnected. Constitutive enhancement of a metabolite can therefore alter carbon–nitrogen balance, vegetative growth, flowering, fruit development, or yield.

A well-defined example is the targeted activation of glutamate decarboxylase to increase γ-aminobutyric acid (GABA) accumulation. The proteins encoded by *SlGAD2* and *SlGAD3* contain C-terminal autoinhibitory domains that restrict enzymatic activity. CRISPR-Cas9-mediated introduction of premature stop codons immediately upstream of these domains generated C-terminally truncated enzymes with increased activity [31]. Selected *SlGAD3*-edited lines accumulated approximately 7- to 15-fold more GABA in red-stage fruit than wild-type plants [31].

The agronomic consequences depended strongly on the targeted paralogue. C-terminal activation of *SlGAD2* was associated with abnormally high GABA accumulation in leaves, reduced plant growth, impaired flowering in some allelic classes, and lower fruit production [31]. In contrast, C-terminal activation of *SlGAD3*, which is expressed more strongly in fruit, increased fruit GABA accumulation without decreasing flowering or fruit yield in the evaluated lines [31]. Closely related paralogues with comparable enzymatic activities therefore produced different whole-plant outcomes because of differences in their expression patterns.

This finding illustrates an important strategy for managing metabolic trade-offs. When several paralogues encode similar biochemical activities, the paralogue whose expression is concentrated in the harvested organ may be a safer target than one with broad vegetative expression. When a suitably tissue-biased paralogue is unavailable, editing a fruit-specific regulatory element may provide greater biological control than constitutive activation of the enzyme.

Carotenoid engineering provides another example of allele- and pathway-dependent effects. Simultaneous Target-AID editing of *SlDDB1, SlDET1*, and *SlCYC-B* generated multiple substitution combinations with different carotenoid profiles [26]. Two independently derived, transgene-null T_1_ lines carrying substitutions at all three loci showed different responses. One line accumulated approximately twice as much lycopene as the wild type, whereas the other exhibited an increase of approximately 30% [26]. The lines also differed in their β-carotene responses [26].

The edited populations displayed high-pigment-associated characteristics, including dwarf growth, purple roots, and darker green fruit [26]. Alterations in photomorphogenesis and plant development therefore accompanied changes in carotenoid accumulation. Furthermore, researchers did not recover homozygous indel alleles of *SlDDB1* [26]. The authors interpreted this result as suggesting that complete disruption of *SlDDB1* could be strongly deleterious or lethal, although they did not demonstrate lethality directly [26]. Partial-function substitutions or fruit-restricted regulatory alleles may therefore be more suitable than complete knockout for such pleiotropic targets.

Nutritional improvement should be evaluated using a pathway-wide framework. Measurement of the target metabolite alone cannot establish agronomic benefit. Studies should quantify related metabolites, pathway precursors and products, fruit colour, ripening, vegetative growth, flowering, fruit set, seed traits, and yield. Broader metabolomic analysis is particularly important when an edited enzyme participates in central carbon or nitrogen metabolism.

### 4.3. Disease Resistance Versus Growth and Productivity

Disease-resistance breeding frequently encounters a potential defence-growth trade-off. Constitutive immune activation can divert resources from growth and reproduction, whereas disruption of a susceptibility gene may interfere with its normal physiological functions. Suitable targets are therefore susceptibility factors whose modification restricts pathogen exploitation without producing unacceptable developmental or productivity costs. Loss-of-function editing of *SlMlo1* generated resistance to tomato powdery mildew [23]. Under the conditions evaluated, the edited plants were morphologically similar to wild-type plants and produced a comparable harvested fruit weight [23]. These findings support the potential utility of *SlMlo1* loss of function but do not establish complete agronomic neutrality across genetic backgrounds, environments, or pathogen pressures.

Disruption of *SlDMR6-1*, which encodes a salicylic acid 5-hydroxylase involved in salicylic acid homeostasis, produced a broader resistance spectrum. The *Sldmr6-1* mutants exhibited increased resistance to the bacterial pathogens *Pseudomonas syringae* pv. tomato, *Xanthomonas gardneri*, and *Xanthomonas perforans*, the oomycete *Phytophthora capsici*, and the powdery mildew fungus *Pseudoidium neolycopersici* [32]. Resistance was associated with increased salicylic acid accumulation and enhanced transcriptional activation of immune responses [32].

Under the tested laboratory conditions, the mutants did not display an evident growth penalty despite transcriptomic changes involving immune activation and suppression of several developmental and photosynthetic processes [32]. Field evaluation under naturally occurring *Xanthomonas* pressure produced a more nuanced outcome. The mutants showed moderate reductions in plant height, internode length, leaf size, and fruit size, including a lower yield of extra-large fruit, but total marketable yield was not significantly different from that of wild-type plants [32]. Because healthy, pathogen-free plants could not be evaluated under those field conditions, the field performance of uninfected *Sldmr6-1* plants remains unresolved [32]. The available evidence therefore supports broad-spectrum resistance and preservation of total marketable yield under the reported disease-affected field conditions, but it does not demonstrate complete agronomic neutrality.

Both *SlDMR6-1* and *SlDMR6-2* converted salicylic acid to 2,5-dihydroxybenzoic acid in biochemical assays [32]. However, *SlDMR6-1* was pathogen-responsive and broadly expressed, whereas *SlDMR6-2* expression was concentrated in flowers and immature fruit and was not induced by the tested pathogens [32]. The authors therefore proposed that duplication was followed by subfunctionalization, with *SlDMR6-1* regulating salicylic acid during pathogen infection in vegetative tissues and *SlDMR6-2* contributing to salicylic acid balance in reproductive organs [32]. Expression and biochemical evidence support the proposed reproductive specialization of *SlDMR6-2*, but it has not been fully established as a breeding outcome.

Targeting the pathogen-responsive *SlDMR6-1* paralogue may preserve reproductive functions more effectively than disrupting both paralogues, but this possibility requires direct reproductive and yield testing. Disease-resistance edits should be evaluated under pathogen-free and infected conditions using measurements of pathogen growth, disease severity, defence-gene expression, salicylic acid and related hormones, vegetative biomass, flowering, fruit set, fruit-size distribution, total marketable yield, and fruit quality. Resistance should also be examined against pathogens with contrasting lifestyles because increased salicylic-acid-associated defence may not improve resistance to every pathogen class.

### 4.4. Heat Resilience Versus Fruit Set, Seed Production, and Fruit Quality

High temperature reduces tomato productivity largely by impairing pollen development, fertilization, and fruit set. Genome editing can address this constraint by enabling fertilization-independent fruit development or protecting reproductive sink strength. These strategies must balance stable fruit production against seed formation, fruit quality, hormonal pleiotropy, and performance under non-stress conditions. Loss of *SlAGAMOUS-LIKE 6 (SlAGL6*) function produces facultative parthenocarpy.

Researchers initially identified the causal allele in an ethyl methanesulfonate-mutagenized population, and independently generated CRISPR-Cas9 loss-of-function alleles confirmed that *SlAGL6* disruption caused the phenotype [33]. Under high-temperature conditions that strongly impaired fertilization-dependent fruit set, *Slagl6* mutants produced seedless fruit of normal or comparable weight and shape [33]. The mutants did not exhibit homeotic abnormalities, pollen viability was unaffected, and sexual reproductive capacity was maintained [33]. Because the phenotype is facultative, the plants can produce seeded fruit when fertilization succeeds [33].

Nevertheless, seedlessness remains the expected outcome when fruit develops parthenocarpically. The suitability of *SlAGL6* loss of function therefore depends on whether seedless fruit is commercially desirable and whether the edited genotype must serve as a parental line in hybrid-seed production. Performance should also be verified after introducing the mutation into elite cultivars and under the horticultural conditions intended for commercial production.

A more recent strategy involved regulatory editing of the *THERMOSENSITIVE PARTHENOCARPY 4* locus, which comprises two linked genes: *TSP4a*, encoding the auxin-response repressor *IAA9*, and *TSP4b*, encoding the transcriptional regulator *AINTEGUMENTA* [34]. Natural regulatory polymorphisms reduced the heat-induced expression of these genes and promoted parthenocarpic fruit development without broadly increasing auxin responses throughout the plant [34].

Complete CRISPR-Cas9 knockout of *TSP4a/SlIAA9* produced strong parthenocarpy under both control and heat-stress conditions, along with broader auxin-associated developmental effects [34]. To obtain a more restricted phenotype, researchers edited open-chromatin regions in the *TSP4a* promoter and the *TSP4b* 3′ untranslated region to reduce rather than eliminate gene expression [34]. Selected regulatory-edited alleles increased parthenocarpic fruit production and improved fruit-bearing ability under heat stress without the broad auxin-related pleiotropic effects associated with complete *SlIAA9* loss of function [34]. Fruit fresh weight and sugar content were maintained, and yield improved under the greenhouse heat-stress conditions evaluated [34]. These conclusions should remain limited to the tested alleles, genetic background, traits, and greenhouse environments.

Prime editing of cell-wall-invertase promoters provides a mechanistically distinct approach. Insertion of a 10-bp heat-shock element enabled heat-responsive upregulation of cell-wall-invertase genes and improved carbon allocation to developing fruit [29]. In tomato cultivar Ailsa Craig, edited lines produced an approximately 33% greater yield per plot than heat-stressed controls without detectable penalties in the fruit-quality parameters reported by the authors [29]. Because target-gene activation was heat-responsive rather than constitutive, the strategy was designed to reinforce reproductive sink strength during heat stress while limiting unnecessary perturbation under non-stress conditions.

The *SlAGL6, TSP4*, and cell-wall-invertase examples therefore represent three different approaches to heat-resilient productivity: facultative parthenocarpy through loss of a reproductive regulator, quantitative and heat-dependent tuning of auxin signalling, and stress-responsive reinforcement of reproductive sink strength. The optimal approach depends on whether seedlessness is commercially acceptable, whether the edited genotype must function as a seed parent, and whether normal reproductive and source–sink relationships must be preserved under non-stress conditions.

### 4.5. Plant Architecture Versus Fertility and Yield

Plant architecture influences planting density, light interception, inflorescence structure, flower production, mechanical harvesting, fruit distribution, and reproductive success. Architectural alleles can improve productivity but may also cause excessive branching, floral abnormalities, reduced fertility, or uneven fruit development.

A well-defined example involves negative epistasis between *JOINTLESS2* (*J2*) and *ENHANCER OF JOINTLESS2* (*EJ2*). A weak *ej2* domestication allele had little effect on its own. In contrast, a mutation in its MADS-box paralogue *J2* was subsequently used to eliminate the fruit-pedicel abscission zone required for mechanical harvesting [16]. Combining the alleles produced excessive inflorescence branching, reduced fertility, and lower productivity [16].

Natural and CRISPR-generated alleles of these MADS-box genes produced a continuum of inflorescence complexity [16]. Alleles with intermediate functional strength moderated excessive branching and enabled higher-yielding hybrids while retaining useful harvesting characteristics [16]. This example demonstrates that complete loss of function is not necessarily the most useful breeding outcome. Intermediate allelic strength can provide a more favourable balance among inflorescence branching, flower number, fertility, and fruit production.

Direct repair of the deleterious domesticated *SSP2* allele provides a complementary strategy for modifying plant architecture. The domesticated allele reduced the DNA-binding activity of the *SSP2* transcription factor, and adenine base editing restored the favourable nucleotide state [13]. The repaired plants exhibited more compact growth and earlier fruit production [13]. Because *SSP2* functions partially redundantly with *SSP*, the effect of the repaired allele depended on the sensitized genetic background produced during domestication [13].

Multiplex de novo domestication further demonstrates the importance of coordinating architectural and reproductive alleles. Editing several domestication-associated loci in wild tomato backgrounds produced compact architecture and altered flowering, fruit number, fruit size, and nutritional characteristics [7,24]. However, the resulting phenotypes reflected the combined effects of multiple alleles rather than establishing the independent contribution of each edit. Single-edited and intermediate genotypes remain necessary for determining whether a multiplex combination contains favourable additive effects or concealed negative epistasis.

Architectural editing should therefore be evaluated quantitatively using branch number, internode length, inflorescence branching, flower number, floral fertility, fruit set, fruit distribution, harvest index, and total yield. Greenhouse cultivation, open-field production, high-density planting, and mechanical harvesting may require different optimal allelic strengths.

### 4.6. General Principles for Editing Trait Trade-Offs

**Figure 2:**
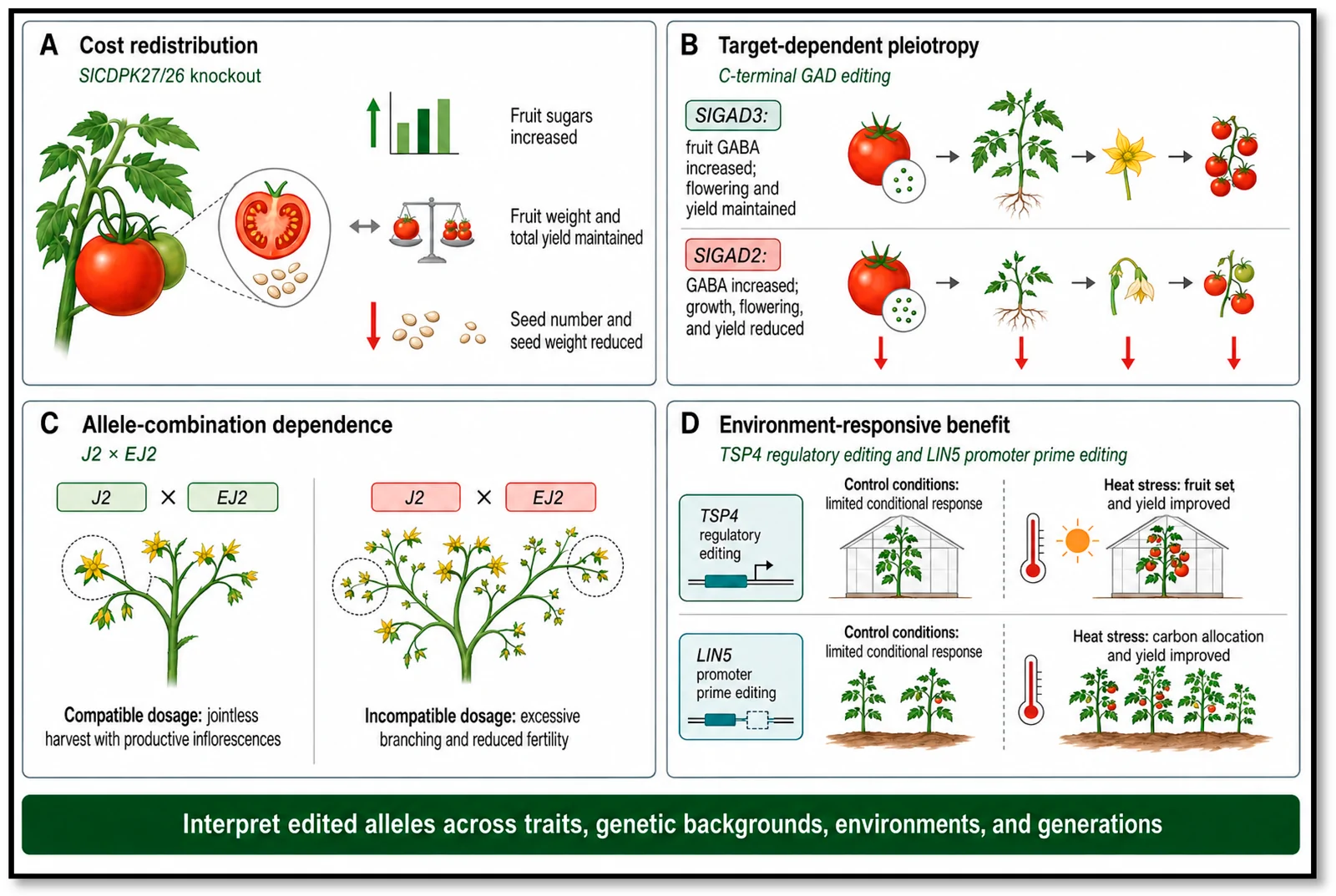
Context-dependent outcomes of genome editing in major tomato trait trade-offs. (**A**) Editing *SlCDPK27* and *SlCDPK26* increased fruit sugar concentrations and maintained fruit weight and total yield but reduced seed number and seed weight. The green upward arrow denotes an increase, the grey bidirectional arrow denotes maintenance, the red downward arrow denotes a reduction, and the dashed connector lines identify the enlarged fruit and seed view. (**B**) Editing *SlGAD3* and *SlGAD2* produced contrasting nutritional and agronomic outcomes associated with their expression patterns. Pale-green and pale-salmon boxes distinguish the *SlGAD3* and *SlGAD2* outcomes, respectively; black arrows link each edited target to the depicted whole-plant, floral, and fruit outcomes, while red downward arrows denote reductions in the corresponding traits. (**C**) *J2–EJ2* interactions demonstrate allele-dosage and epistatic effects on inflorescence architecture and fertility. Pale-green boxes indicate the compatible allele combination, pale-salmon boxes indicate the incompatible combination, and dotted circles highlight representative inflorescence phenotypes. (**D**) Regulatory editing of *TSP4* and prime editing of the *LIN5* promoter illustrate conditional improvements in reproductive performance and yield under heat stress. Pale-green and pale-blue boxes identify the *TSP4* and *LIN5* editing strategies, respectively; vertical dashed lines separate control and heat-stress conditions, and the thermometer and sun symbols denote elevated temperature. Colours are panel-specific and do not represent a common quantitative scale.

Across these examples, the most favourable outcomes occurred when an edit’s strength and expression domain matched the target’s biological function. Editing the fruit-biased *SlGAD3* increased fruit GABA without the growth, flowering, and yield penalties associated with comparable modification of the more broadly expressed *SlGAD2* under the reported conditions [31]. Conditional heat-responsive alleles likewise improved reproductive performance or yield under elevated temperature while limiting unnecessary constitutive activation [29,34]. Graded regulatory or partial-function alleles may therefore be preferable to complete knockouts in dosage-sensitive pathways [8,16,26,34]. Nevertheless, increased fruit sugar without a detected reduction in fruit weight or total yield was accompanied by reduced seed production [12], showing that alleviating one trade-off can shift the biological cost to another trait. Epistasis and genetic background may further alter the outcome of an otherwise favourable allele [10,16]. Figure 2 illustrates selected context-dependent editing outcomes, whereas Table 1 summarises additional examples and their remaining biological limitations.

## 5. Experimental Validation of Trade-Off Mitigation

Improving the intended trait alone does not demonstrate that a genome-edited tomato line has overcome a biological trade-off. The edited phenotype must be reproducible, the anticipated competing traits must remain within acceptable limits, and the allele must be genetically stable under cultivation-relevant conditions. Validation should therefore combine causal analysis of the target, complete characterization of the edited locus, comparison of several edited alleles, multi-trait phenotyping, inheritance testing, and evaluation across genetic backgrounds and environments. These requirements are particularly important when the target regulates several developmental or metabolic processes, because changes in yield, reproduction, fruit composition, or stress responses may be less immediately apparent than the selected phenotype.

### 5.1. Establishing the Causal Basis of the Editing Target

Target selection should begin with evidence that the proposed gene, regulatory sequence, or nucleotide variant contributes directly to the trait relationship under investigation. Genetic association, differential expression, and pathway membership can identify candidate loci, but they cannot by themselves distinguish a causal variant from linked polymorphisms, background-specific modifiers, or structural variation. The molecular effect of the existing allele must therefore be determined before choosing knockout, cis-regulatory editing, or precise allele replacement.

The ripening inhibitor (*rin*) locus illustrates why the molecular identity of a classical mutant allele must be distinguished from complete gene loss. CRISPR-Cas9-induced loss-of-function alleles of *RIN* did not reproduce the complete non-ripening phenotype of the classical *rin* mutant. Fruits carrying the edited alleles initiated several ripening processes, whereas the classical mutation encodes a RIN–MACROCALYX fusion protein that actively represses ripening-associated transcription [35]. Thus, the phenotype of a classical mutant cannot automatically be interpreted as the consequence of simple loss of gene function.

CRISPR-Cas9 analysis of *AP2a*, *NAC-NOR*, *FUL1*, and *FUL2* reached a similar conclusion. The *NAC-NOR* null allele produced a substantially milder ripening phenotype than the classical *nor* mutant. Single and combined mutations of *FUL1* and *FUL2* revealed partially redundant functions during ripening and an additional contribution of *FUL2* to early fruit development [36]. Further examination of the *nor* locus showed that the truncated NOR186 protein encoded by the classical allele retained promoter-binding capacity but lacked normal transcriptional activation activity and interfered with the wild-type protein. By comparison, *NAC-NOR* null mutants showed less severe reductions in ethylene production, carotenoid accumulation, and fruit softening [37]. These studies show that null, hypomorphic, gain-of-function, and dominant-negative alleles at the same locus can produce different phenotypes.

Causal analysis must also include structural variation. Comparison of diverse tomato genomes revealed extensive copy-number, presence–absence, and other structural variants associated with gene expression, fruit size, flavour-related chemistry, and harvest traits. Experimental reconstruction of selected variants further showed that structural changes and interactions among loci can contribute directly to agronomic phenotypes [20]. Target discovery should therefore consider local haplotype and structural context rather than relying exclusively on single-nucleotide differences relative to one reference genome.

A suitable target model should explain how the locus influences both the desired phenotype and its likely biological cost. It should also identify the tissues and developmental stages in which the target functions and justify whether complete knockout, partial reduction of activity, regulatory modification, or nucleotide replacement is appropriate. When causality remains uncertain, knockout, complementation, allele-repair, or expression-modulation experiments should precede introduction of the edit into elite material.

### 5.2. Allelic Series and Experimental Comparators

One edited genotype rarely provides enough evidence to demonstrate trade-off mitigation. Independently regenerated events carrying the same intended allele are needed to determine whether the phenotype is consistently associated with that allele rather than with an unrelated mutation or tissue-culture variation. When different editing products are likely to retain different levels of gene activity, retain several alleles for comparison instead of selecting only the line with the strongest initial phenotype. For dosage-sensitive or pleiotropic genes, an allelic series may identify an activity level that improves the target trait without producing the penalties associated with complete loss of function. Such a series may contain frameshift mutations, in-frame deletions, promoter variants, upstream open-reading-frame modifications, or defined nucleotide substitutions. However, neither an edit’s physical size nor its predicted molecular severity reliably determines the resulting phenotype. This limitation was demonstrated by editing a conserved cis-regulatory region in the tomato *KLUH promoter*. Twenty-one promoter alleles containing different insertions and deletions were generated, but only five consistently increased fruit weight and reduced the proportion of small fruits across experiments. Neither allele type nor the measured change in *KLUH* transcript abundance reliably predicted the fruit-weight phenotype [38]. Cis-regulatory alleles must therefore be evaluated through replicated phenotyping rather than ranked solely by deletion size or expression level. For redundant genes, comparisons should include single mutants, intermediate combinations, and the complete multiplex genotype. This design separates functional redundancy from additive or epistatic interactions. The requirement is particularly relevant in tomato because combinations of individually favourable domestication alleles can produce negative epistasis and undesirable plant architecture [16]. Evaluation of only the final multiplex genotype would not reveal which combination caused the adverse phenotype.

Experimental controls should include the unedited recipient genotype, independently generated edited events, relevant single and intermediate mutant combinations, and, when available, a naturally occurring or conventionally bred favourable allele. Regenerated but unedited plants can help identify tissue-culture-associated phenotypes. When transformation integrates an editing construct, compare transgene-free edited segregants with non-edited siblings from the same family. Complemented or precisely repaired lines provide especially strong evidence when the causal interpretation of a target remains uncertain.

### 5.3. Molecular Characterization of Edited Events

Molecular validation must establish the complete identity of the edited allele. Short amplicon sequencing is useful for initial screening, but it may not detect large deletions, rearrangements, copy-number changes, vector fragments, partial donor integration, or complex allele mixtures. The analytical methods selected for advanced lines should reflect the editing system and the expected product. Deep amplicon sequencing can quantify mosaicism and low-frequency alleles. In contrast, structurally complex events may require long-range PCR, junction sequencing, copy-number analysis, long-read sequencing, or whole-genome sequencing.

Geminiviral replicons were used to insert a promoter upstream of the tomato *ANT1* locus. More than two-thirds of the examined targeted insertions had the intended sequence, selected modifications were inherited in a Mendelian manner, and no persistent extrachromosomal replicons were detected in the plants examined [39]. However, recovering precise products in some events does not remove the need to screen every advanced line for imprecise junctions, one-sided recombination, unintended donor sequences, or residual vector fragments.

A related CRISPR-Cas9 geminiviral system repaired a 281-bp deletion in the tomato *crtiso* allele through homologous recombination. Repaired alleles were detected in 25% of the transformed plants under the conditions used, without requiring a selectable marker linked to the repaired locus [40]. This result established that direct allele repair is feasible in tomato, but the reported frequency should not be generalized across loci. Homology-directed repair depends on the target sequence, donor design, delivery system, genotype, regeneration procedure, and culture conditions.

A CRISPR/LbCas12a geminiviral multi-replicon system increased homology-directed repair approximately threefold relative to the Cas9 single-replicon comparator used in that study. It produced a stable, replicon-free, marker-free *HKT1;2* replacement allele, and editing performance varied with temperature and light conditions during culture [41]. These results demonstrate that the editing architecture and regeneration environment can influence both the frequency and the composition of recovered alleles.

CRISPR-Cas9-induced double-strand breaks have also stimulated recombination between homologous tomato chromosomes at *PSY1*. Molecular analysis detected homologous recombination products, including germinally transmitted events, when the intact homolog served as the repair template [42]. Such recombination could help separate linked favourable and unfavourable alleles, but breeding applications require confirmation of recombination boundaries, germline transmission, and chromosome integrity.

Gene replacement at the *ALC* locus provides another example of why primary regenerated plants are insufficient for final validation. The reported replacement frequency in T_0_ plants was 7.69%, whereas homozygous, T-DNA-free *alc* progeny with improved storage performance were recovered after segregation in the T_1_ generation [43]. Editing success in T_0_ plants must therefore be followed by fixation of the intended allele, removal of the editing construct, and phenotypic confirmation in stable descendants.

Molecular tests should match the editing method. Nuclease-induced alleles require confirmation of zygosity, mosaicism, deletion boundaries, and possible target-site rearrangements. Base-edited lines should be screened for the intended substitution, bystander substitutions, and local insertions or deletions. Prime-edited lines require analysis of the complete intended product, partial editing products, scaffold-derived insertions, indels, and heterogeneous alleles because their frequencies differ among prime-editing systems and target sites [25,26,27,28,29,30].

DNA-free editing refers to delivery of genome-editing reagents without a DNA vector, for example, as preassembled CRISPR-Cas ribonucleoprotein complexes. In contrast, transgene-free refers to the final regenerated plant or its progeny in which no stably integrated editing construct is detected; a transgene-free plant may therefore originate from DNA-based transformation followed by segregation. Thus, DNA-free describes the delivery process, whereas transgene-free describes the recovered plant’s molecular status. Similarly, Cas9-free, T-DNA-free, marker-free, and replicon-free should refer only to the specific component demonstrated to be absent. DNA-free ribonucleoprotein delivery reduces the possibility of construct integration but does not remove the need to confirm target-site integrity, zygosity, and genetic uniformity [14]. Whole-genome sequencing of the transgene-free Tomelo line did not detect guide-associated mutations outside the intended target under the sequencing and analytical conditions used [23]; this finding should not be generalized to all genome-edited tomato lines.

### 5.4. Multi-Trait and Developmentally Resolved Phenotyping

Evidence of trade-off mitigation requires concurrent evaluation of the intended benefit and its biologically plausible costs. Therefore, the primary breeding objective, competing traits, sampling stages, and acceptable performance ranges should be defined before phenotyping. Measurements should be obtained from the same plants and harvests, because evaluating the favourable phenotype and the anticipated penalty in separate experiments may obscure trait covariance and genotype-by-environment interactions. Comparisons should include the unedited recipient, independently generated edited events, appropriate segregating controls, and, when available, a conventionally bred genotype expressing a comparable favourable phenotype.

The measurement panel should reflect the function of the edited locus rather than applying an identical set of traits to every genotype. Yield-related evaluation should distinguish fruit number, individual fruit mass, total yield, marketable yield, harvest timing, and defective-fruit frequency. Fruit-quality analysis may require measurements of individual sugars, organic acids, volatile compounds, carotenoids, firmness, seed characteristics, and sensory responses. Soluble-solids content alone cannot represent tomato flavour because consumer preference depends on interacting contributions from sugars, acids, volatile compounds, and texture [18]. Broader metabolic profiling is particularly important for edits involving transcription factors, hormone-signalling components, transporters, or metabolic enzymes with extensive downstream effects [19].

Pathway-specific experiments should connect molecular effects with agronomic performance. Heat-responsive editing of the LIN5 promoter should be evaluated through fruit set, fruit number, fruit mass, yield, sugar composition, and vegetative growth under both control and heat-stress conditions [29]. GABA-enriched genotypes should be assessed for GABA and related metabolites, along with fruit development, seed formation, plant growth, flowering, and yield [31]. Disease-resistance lines require defined pathogen isolates, suitable inoculation controls, quantitative disease measurements, and agronomic assessment under infected and non-infected conditions. For example, resistance associated with SlDMR6-1 disruption should be evaluated alongside growth, fruit size, yield, and fruit quality in the intended production environment [32].

Edits affecting reproductive development or ripening require similarly integrated measurements. The evaluation of SlAGL6-mediated facultative parthenocarpy should include flower morphology, pollination response, fruit set, fruit mass, seed production, and marketable yield [33]. Heat-resilient fruit set associated with TSP4 should be assessed using pollen viability, flower retention, fruit number, fruit mass, sugar concentration, and final yield rather than inferring it solely from molecular stress responses [34]. Ripening-related alleles require simultaneous measurement of ethylene production, colour development, carotenoid accumulation, firmness, postharvest behaviour, flavour-associated chemistry, and decay susceptibility. Studies of *RIN* and *NAC-NOR* demonstrate that these ripening components can be affected to different degrees; consequently, colour development or firmness measured at one time point does not establish normal ripening or selective improvement of shelf life [35,36,37].

Phenotyping should also account for developmental stage and tissue identity. Whole-fruit or bulk-pericarp measurements may conceal localized responses in the epidermis, placenta, locular tissue, vascular tissue, or seeds. Transcriptome analyses have demonstrated extensive tissue- and stage-specific gene expression during tomato fruit development [44,45]. Molecular and biochemical measurements should therefore examine the tissues and developmental intervals in which the edited target is expected to function. When an edit changes flowering, fruit set, or ripening rate, comparisons at both equivalent chronological ages and equivalent physiological stages may be necessary to distinguish developmental delay from a stable change in the final phenotype.

Postharvest evaluation should distinguish selective improvement in firmness or storage integrity from generalized suppression of ripening. RNA interference of tomato pectate lyase reduced fruit softening without preventing major ripening-associated colour and compositional changes under the reported conditions. Although this was not a genome-editing experiment, it provides biological evidence that a downstream cell-wall target can modify softening more selectively than an upstream ripening regulator [46]. Genome editing of the *FIS1* locus increased fruit firmness and shelf life through changes in cutin and wax deposition without detectable alterations in fruit weight, colour, ripening time, or the measured sugar and acid contents in the materials evaluated [47]. Postharvest assessment should therefore include instrumental firmness, water loss, shrivelling, cracking, decay, colour, chemical composition, and storage integrity in fruits collected at equivalent physiological stages.

Reproductive and vegetative phenotypes should be evaluated together when the target participates in hormone signalling or developmental regulation. Disrupting *SlIAA9* generated parthenocarpic fruits but also altered leaf architecture, showing that a reproductive benefit can be accompanied by a vegetative phenotype [48]. Subsequent work in elite tomato materials further showed that transformation responsiveness, recovery of stable homozygous progeny, construct segregation, and seed production affect the practical use of *SlIAA9* alleles in breeding [49]. Evaluation should therefore connect the exact edited genotype with flower development, pollination response, fruit formation, seed production, vegetative architecture, yield, and maintenance of stable edited lines.

### 5.5. Linking Allele Identity, Inheritance, and Phenotype

Molecular characterization establishes the sequence of an edited allele, whereas genetic validation determines whether that allele is stably inherited and consistently associated with the observed phenotype. The target-site sequence, zygosity, allelic composition, editing-construct status, and phenotype should therefore be recorded for each plant and its descendants. Primary regenerants may be homozygous, heterozygous, biallelic, or chimeric, and different editing products may produce phenotypes of different strengths. Combining all regenerated plants into a single “edited” group can consequently obscure allele-specific effects.

Molecular characterization of 361 T_0_ tomato plants targeting 63 immunity-associated genes demonstrated this variation. Researchers detected target-site mutations in 245 plants, representing 68% of the experimental collection. The recovered plants included homozygous, heterozygous, biallelic, and chimeric genotypes, and 87% of the affected sequences contained insertions or deletions shorter than 10 bp. Most mutations were transmitted to subsequent generations, but some progeny also showed new target-site alleles while active editing components remained present [50]. A primary regenerated plant should therefore not be assumed to contain a genetically uniform or fixed allele.

Genotyping should continue after segregation, and final phenotyping should use descendants in which the intended allele is fixed. When construct removal is required for the breeding objective, segregation of the editing construct should be confirmed rather than inferred from the absence of a selectable phenotype. Independently generated events carrying the same allele, edited and non-edited siblings from the same family, and stable progeny provide stronger evidence that the phenotype is associated with the intended allele rather than with mosaicism, tissue-culture variation, or another segregating mutation.

Specificity conclusions must be limited to the material and analytical methods examined. In the same experimental collection, no mutations were detected at 18 predicted off-target sites examined in 144 plants [50]. This result supports the specificity of those guide-target combinations at the candidate sites tested, but it does not demonstrate the absence of every possible genome-wide mutation. Molecular measurements should also test the proposed biological mechanism. Promoter edits should be linked to target-gene expression in the relevant tissues and developmental stages, whereas metabolic edits should be connected to appropriate pathway substrates, intermediates, and products. Such measurements strengthen causal interpretation but cannot substitute for multi-trait phenotyping.

An allele should be considered genetically validated only when its molecular identity is unambiguous, its inheritance is stable, and its phenotype is reproducible in independent events or segregating families. Evidence of trade-off mitigation additionally requires an acceptable combination of the intended and competing traits relative to both the unedited recipient and an appropriate breeding comparator. If the predicted agronomic penalty has not been measured, the result should be described as modification of the target trait rather than demonstrated mitigation of the trade-off.

### 5.6. Genetic Background and Environmental Stability

An edited allele characterized in one tomato cultivar should not be assumed to produce the same phenotype in another. Recipient backgrounds may differ in promoter sequences, paralogue activity, structural variation, modifier loci, and domestication haplotypes. The negative epistasis between *J2* and *EJ2* demonstrates that the effect of an otherwise useful allele can change when combined with a pre-existing domestication allele [16]. Structural variants affecting gene dosage, regulatory activity, and local haplotype composition provide an additional source of background-dependent phenotypic variation [20]. Genetic background should therefore be treated as an experimental variable rather than as a passive property of the recipient cultivar.

Near-isogenic materials are preferable for separating the effect of an edited allele from genome-wide background differences. After the initial transformation in a model genotype, the phenotype should be confirmed after reconstruction or introgression into the intended elite cultivar. Results obtained only in ‘Micro-Tom’ can provide valuable functional evidence but do not establish commercial agronomic performance. If broad applicability is claimed, the same allele should be evaluated in representative genetic backgrounds relevant to the intended market classes and production systems.

Environmental validation should reproduce the conditions under which both the proposed benefit and its associated cost are expected to occur. Temperature, water availability, salinity, nutrient supply, radiation, pathogen pressure, protected cultivation, and open-field production can alter the intended phenotype and competing traits. Evaluation should progress from controlled experiments to replicated greenhouse or field trials across relevant environments and, when feasible, more than one growing season.

The importance of such testing is illustrated by a tomato multi-parent advanced-generation intercross population evaluated across 12 environments, including optimal, water-deficit, salinity, and heat-stress conditions. Significant genotype-by-environment interactions were detected for every measured trait, and mean performance and phenotypic plasticity had partly distinct genetic determinants [51]. Evaluation of an edited allele should therefore consider both its average performance and the stability of its multi-trait response across environments.

Environmental treatments should be biologically relevant to the proposed phenotype. Heat-resilience alleles require temperatures that disrupt the reproductive or physiological process under investigation, whereas disease-resistance alleles require appropriate pathogen isolates and conditions conducive to infection. Fruit-quality alleles should be evaluated under contrasting cultivation conditions because temperature, radiation, irrigation, and mineral nutrition can alter sugars, acids, pigments, volatile compounds, firmness, and ripening. A trade-off should not be described as broadly mitigated when the favourable effect occurs in only one environment or when a competing trait deteriorates under stress.

**Figure 3:**
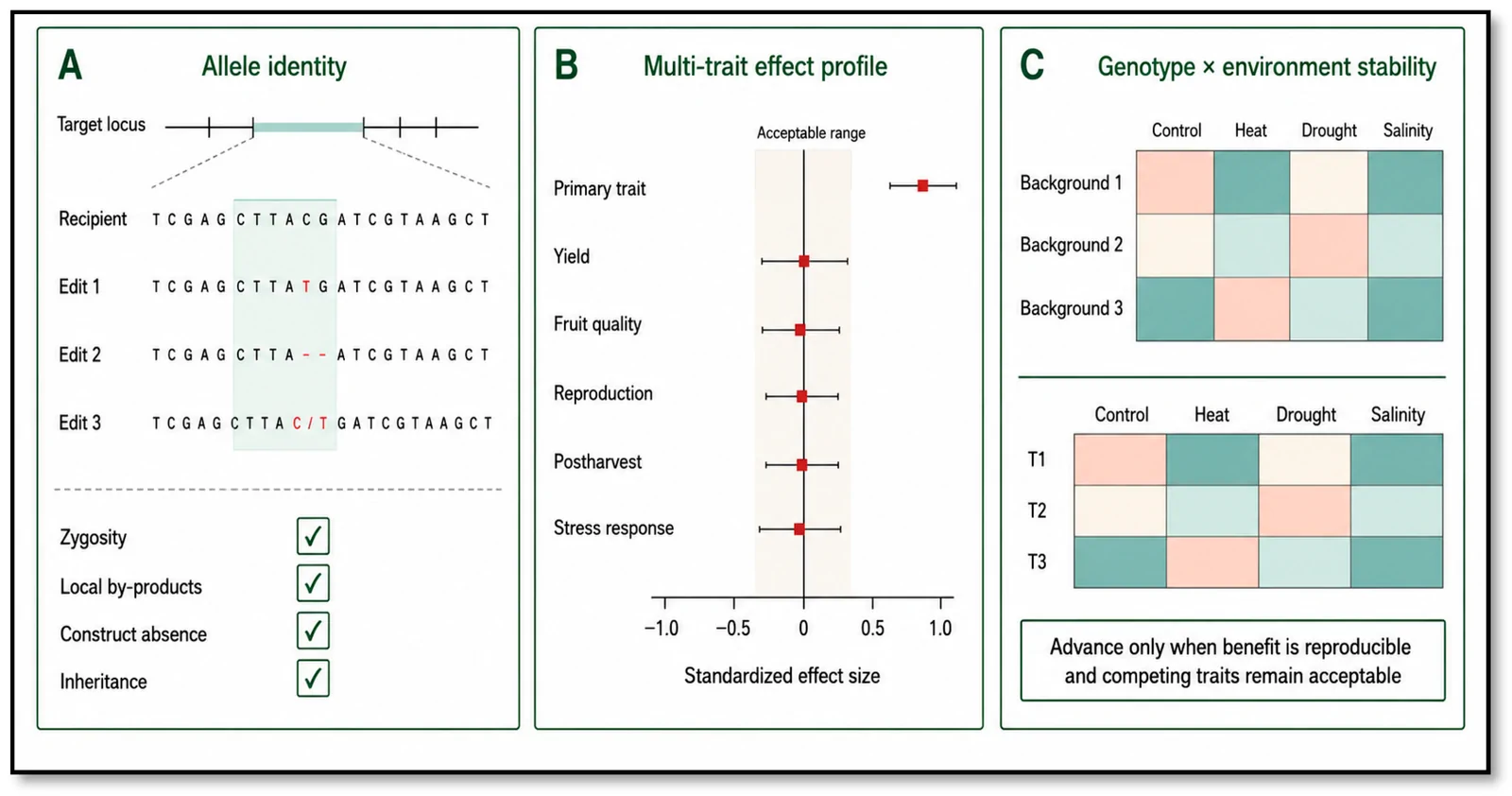
Evidence framework for validating mitigation of trait trade-offs in genome-edited tomato. (**A**) Confirm edit identity, local sequence integrity, zygosity, construct absence where claimed, and stable inheritance. Pale teal shading marks the target interval, red characters or dashes indicate sequence changes, and green check marks indicate confirmed validation criteria. (**B**) Quantify the intended phenotype and biologically plausible competing traits against predefined performance criteria. The beige band denotes the acceptable range, red squares represent illustrative effect estimates, black horizontal lines represent uncertainty ranges, and the vertical line marks zero effect. (**C**) Evaluate phenotypic stability across relevant genetic backgrounds, environments, and generations. Teal, off-white, and salmon cells indicate relatively favourable, neutral, and unfavourable responses, respectively. Effect estimates and heat-map patterns are conceptual and do not represent experimental data.

Evidence of successful mitigation should demonstrate a reproducible improvement in the primary breeding target while agronomic performance, reproductive capacity, fruit quality, and stress responses remain within predefined acceptable ranges. Any limitation to a particular genetic background, environment, developmental stage, or measured endpoint should be stated explicitly. Figure 3 summarises the integrated validation pathway, whereas Table 2 specifies the core evidence and comparisons required to establish trade-off mitigation.

## 6. Breeding Deployment and Regulatory Translation of Edited Tomato Alleles

Translation of an edited allele into a tomato variety requires more than recovery of the intended DNA change. The allele must perform reliably in breeding-relevant germplasm, remain stable across generations, and retain its value under the production conditions for which the variety is intended. Deployment also requires accurate product identification, appropriate regulatory documentation, seed-system management, and communication that distinguishes experimentally demonstrated benefits from untested assumptions.

### 6.1. Transfer of Edited Alleles into Breeding-Ready Germplasm

Recovery of a tomato plant carrying the intended edit does not by itself establish breeding value. The allele must be incorporated into genetically stable material that retains acceptable yield, fruit quality, reproductive performance, environmental adaptation, and seed-production capacity. This requirement is particularly important when editing is first performed in an easily transformable experimental genotype, because the phenotypic effect may change after transfer into fresh-market, processing, cherry-tomato, rootstock, or locally adapted backgrounds.

Multiplex editing of coding sequences, cis-regulatory regions, and an upstream open reading frame associated with plant architecture, flowering, fruit development, and ascorbic acid biosynthesis modified four wild *Solanum pimpinellifolium* accessions.

The edited wild accessions retained the bacterial-spot resistance or salt tolerance evaluated in their respective parental backgrounds [24]. In an independent study, editing six domestication-associated loci in *S. pimpinellifolium* substantially altered plant architecture, fruit size, fruit number, and nutritional composition [7]. These studies demonstrate that genome editing can accelerate the introduction of domestication traits into wild germplasm. They do not, however, establish that the resulting materials are immediately suitable for commercial cultivation. Wild backgrounds may retain undesirable alleles affecting flavour, texture, ripening, harvestability, shelf life, or regional adaptation.

When the causal allele is known, recreating the edit directly in an elite genotype can avoid transferring the extended donor haplotype that accompanies conventional introgression. Nevertheless, the reconstructed allele’s effect may depend on paralogues, interacting loci, promoter variants, structural variation, and source-sink relationships in the recipient genome. Therefore, the same edit should be tested in representative breeding backgrounds rather than assuming its effect will remain unchanged across cultivars.

If direct editing of an elite parent is limited by transformation or regeneration efficiency, transfer the allele by crossing an edited donor with the target parent. Subsequent generations should be selected for the intended edit, recovery of the elite genetic background, and removal of the editing construct. The resulting material should be evaluated as a specific combination of the edited allele and recipient genome rather than as a phenotype determined by the edited locus alone.

### 6.2. Deployment of Multiplex and Trait-Stacked Genotypes

Multiplex editing is useful when a tomato ideotype requires coordinated modification of architecture, flowering, fruit load, composition, or stress responses. However, the phenotype of a multiplex genotype cannot be predicted simply by combining the effects reported for its component alleles. Functional redundancy, dosage effects, additive interactions, and epistasis can strengthen the intended phenotype or produce previously undetected penalties.

Mutations affecting *SELF PRUNING (SP)*, *SELF PRUNING 5G (SP5G)*, and the ERECTA-family gene *SlER* were combined to produce compact tomato plants with rapid flowering, shortened internodes, and concentrated fruit production. The edited plants maintained yield under the field conditions examined and were also evaluated in an indoor production system [52]. This study demonstrates that combinations of defined alleles can alter tomato architecture for space-limited cultivation. It does not establish that the same combination will maintain productivity across cultivars, planting densities, seasons, or field-management systems.

Single mutants, informative intermediate combinations, and the complete multiplex genotype should be retained whenever possible. Their comparison distinguishes additive effects from redundancy and epistasis and identifies the component responsible for an undesirable phenotype. This is especially important when plant compactness, flowering time, reproductive termination, and fruit load are modified together.

Agronomic performance should be measured within the intended production system. A compact genotype may increase yield per unit area at high planting density without increasing yield per plant. Evaluation should therefore include marketable yield per unit area, yield per plant, harvest concentration, planting density, fruit uniformity, labour requirements, and resource-use efficiency. Conclusions about yield improvement should specify which of these variables changed.

Not every allele introduced during experimental trait stacking must be retained in the final variety. Unnecessary edits increase molecular-characterization requirements, complicate seed purification, and add segregation and documentation demands. When two combinations provide comparable mitigation of the target trade-off, the combination containing fewer modified loci is preferable for breeding deployment.

### 6.3. Molecular and Phenotypic Identity of the Final Product

A breeding-ready edited line requires a product-identity profile connecting the recovered DNA sequence with the observed phenotype. This profile should establish the complete sequence and zygosity of every intended edit, inheritance of the edited alleles, segregation history, and absence of editing-vector sequences when a transgene-free product is claimed.

In the accelerated-domestication study, researchers selected progeny reported to lack detectable Cas9 sequences and examined predicted off-target sites for six targets using T7 endonuclease I assays and Sanger sequencing [24]. These analyses support characterization of the reported lines, but they should not be interpreted as genome-wide proof that all unintended sequence changes were absent. Conclusions concerning specificity must remain limited to the sites, lines, generations, and analytical methods examined.

A null segregant is a progeny plant in which an integrated editing construct has been removed through segregation while the intended edit is retained. The term confirms loss of the construct only to the extent demonstrated by the molecular assays used. It does not automatically establish the absence of unintended target-site products, off-target mutations, somaclonal variants, or structural changes. Conversely, detecting a sequence variant does not, by itself, demonstrate biological harm. Its genomic position, molecular consequence, inheritance, and phenotypic association must be evaluated.

The importance of connecting molecular identity with whole-plant performance is illustrated by editing tomato glutamate decarboxylase genes. Mutations truncating the C-terminal autoinhibitory regions of *SlGAD2* and *SlGAD3* increased fruit γ-aminobutyric acid accumulation, but the phenotypic consequences differed between the two targets. Truncation of *SlGAD2* was associated with reduced plant growth, flowering, and yield, whereas a selected *SlGAD3* truncation increased fruit GABA without a detectable reduction in flowering or yield under the conditions examined [31]. Related enzymes can therefore produce similar compositional changes but different agronomic costs.

The identity profile for a high-GABA tomato should include the edited sequence, GABA concentration, tissue and developmental stage sampled, fruit number, fruit mass, yield, plant growth, flowering, ripening, soluble solids, acidity, and other compositionally relevant measurements. Evidence that an edit increases a metabolite must remain separate from evidence supporting a physiological or health effect in consumers.

### 6.4. Progression from Experimental Material to a Marketed Variety

The commercial introduction of a high-GABA genome-edited tomato in Japan demonstrated that a nutritionally differentiated tomato could progress from experimental development to market deployment [53]. This example is important for tomato breeding, but market introduction does not independently validate every nutritional, physiological, or health claim associated with increased GABA. Each claim requires evidence and regulatory consideration appropriate to the stated effect.

Commercial translation requires preservation of the intended allele and phenotype during crossing, selection, seed multiplication, and variety maintenance. Molecular markers should distinguish the intended allele from the wild-type sequence and from other editing products recovered at the same target. For multiplex lines, markers are needed for every component allele so that the complete stack can be distinguished from progeny that have lost one or more edits.

The final candidate should be compared with an appropriate commercial cultivar, not only the experimental wild type used during gene-function analysis. This comparison should determine whether the edited line provides a meaningful advantage within the intended production system and whether the initial trade-off has been reduced rather than transferred to another trait. For example, an increase in yield per unit area resulting primarily from closer planting should be separated from changes in yield per plant, fruit quality, crop duration, and production inputs.

Seed multiplication must also preserve genetic purity and phenotypic uniformity. This is particularly important for recessive alleles, multiplex genotypes, and edits associated with altered fertility or seed production. Breeder and foundation seed should be tested for allele identity, zygosity, segregation of the editing construct, and the phenotypic characteristics used to define the variety.

### 6.5. Jurisdiction-Specific Regulatory Classification of Genome-Edited Tomato Products

Regulatory classification depends on the final product’s characteristics rather than the editing platform alone. Consequently, plants produced through CRISPR-Cas nucleases, base editing, prime editing, cis-regulatory editing, or multiplex editing do not have a uniform regulatory status. Relevant factors include the nature and number of sequence changes, the presence of introduced genetic material, the intended trait, and the jurisdiction in which cultivation or marketing is proposed.

In the European Union, Regulation (EU) 2026/1388 [54] distinguishes category 1 and category 2 plants obtained through specified new genomic techniques. Plants that meet the applicable equivalence and trait-eligibility criteria may undergo category 1 verification, while category 2 plants remain subject to GMO authorisation, traceability, and labelling requirements. Herbicide-tolerant plants and plants engineered to produce known insecticidal substances cannot qualify as category 1. The Regulation will apply generally from 17 July 2028; until then, targeted mutagenesis and cisgenesis remain subject to the existing GMO framework. Therefore, the absence of foreign DNA alone is insufficient to establish category 1 status [54].

Japan applies a product- and line-specific notification procedure for eligible genome-edited foods. The Ministry of Health, Labour and Welfare separately records the high-GABA tomato lines 87-17 and 206-4, demonstrating that notification of one edited line does not determine the regulatory status of another line affecting the same gene or biochemical pathway. These records represent notifications under the Japanese procedure for foods produced using genome-editing technology and should not be described as conventional GMO approvals [55].

### 6.6. Consumer Communication and Post-Release Stewardship

Regulatory eligibility does not establish agronomic usefulness or consumer acceptance. An Internet-based survey of 550 respondents in Japan conducted in 2021 found that perceived usefulness and confidence in information were associated with willingness to purchase genome-edited foods. The study also reported limited awareness of genome-editing technology and acknowledged that its online sampling method restricted the generalizability of the results [56]. These findings represent one Japanese survey population and should not be treated as a universal measure of consumer opinion.

Communication should identify the edited trait, the biological purpose of the modification, the evidence supporting the intended benefit, and the limitations of the available data. General statements that genome-edited tomatoes are necessarily transgene-free, nutritionally superior, environmentally beneficial, or free from unintended changes should be avoided. Each property should be established for the specific final line.

Post-release stewardship should preserve the identity and genetic purity of the released allele or allele combination. Unexpected deviations in agronomic performance, fruit quality, reproduction, or seed production should prompt product-specific genotyping and phenotyping. Deployment remains successful only as long as the released variety retains its intended advantage under the production and market conditions for which it was evaluated. Table 3 summarizes the evidence required for breeding deployment, final-product characterization, jurisdiction-specific regulatory determination, and post-release stewardship of genome-edited tomato lines.

## 7. Future Research Priorities for Trade-Off-Aware Tomato Genome Editing

Future research should move beyond demonstrating that a tomato gene can be edited and determine whether a defined allele produces a useful balance among productivity, quality, resilience, and reproduction. Priority should be given to causal variants, graded or environmentally responsive alleles, genotype-accessible delivery systems, and validation across breeding backgrounds and production environments. These advances must be accompanied by evidence standards that distinguish successful editing from successful breeding.

### 7.1. Prioritizing Causal Alleles with Defined Biological Effects

Future target discovery should prioritize genes and variants supported by genetic association, a defined molecular mechanism, and direct phenotypic validation. The *SlCDPK27*/*SlCDPK26* and *SSP2* studies illustrate how mechanistic evidence can distinguish a causal editing target from a correlated candidate [12,13]. Before editing, target definition should specify the causal sequence change, relevant tissue and developmental stage, anticipated benefit, plausible secondary consequences, and appropriate editing strategy. This information is necessary to determine whether to modify the target through complete loss of function, quantitative regulatory editing, or precise allele repair.

### 7.2. Moving from Complete Knockout to Quantitative Allele Engineering

Future editing strategies should move beyond routine use of complete loss-of-function alleles when the target is dosage-sensitive, pleiotropic, or required in particular tissues or developmental stages. Promoter variants, transcription-factor-binding-site modifications, amino-acid substitutions, and tissue- or condition-responsive alleles can provide graded changes in gene activity while preserving essential functions. Improved prime-editing systems have expanded the recovery of precise and heritable sequence changes, but editing performance and unintended local products remain dependent on the target and experimental context [28,30]. Future studies should therefore compare complete knockouts with quantitative regulatory and coding alleles to identify the activity range that improves the breeding trait without imposing unacceptable developmental or agronomic penalties.

### 7.3. Engineering Environment-Responsive Alleles

Environment-responsive alleles may reduce the costs of constitutively activating sink, defence, or stress-response pathways. Insertion of a 10-bp heat-shock element into the *LIN5* promoter produced heat-responsive gene activation and increased yield per plot under the reported heat-stress conditions without a detected reduction in the fruit-quality characteristics examined [29]. Future evaluation should determine the activation threshold, duration, reversibility, and tissue specificity of such responses across cultivars, seasons, and production systems. Performance should also be tested under non-stress conditions and realistic combinations of heat, drought, salinity, and pathogen pressure.

### 7.4. Integrating Structural Variation and Recombination Landscapes into Target Selection

Structural variation can create linkage drag and alter gene copy number, promoter organization, guide-RNA complementarity, and DNA-repair outcomes. Genome assemblies of *Solanum pennellii* and *Solanum cheesmaniae*, together with analysis of 709 recombinant plants, identified sex-dependent crossover patterns and recombination cold spots associated with large inversions and insertion–deletion polymorphisms [57]. Direct editing in an elite cultivar may avoid transfer of an extended undesirable donor haplotype, but only after the causal variant has been distinguished from linked markers. Target design should therefore use sequence and structural-variation information from the intended recipient genotype, particularly when the locus lies within a wild introgression, duplication, inversion, or other structurally variable region.

### 7.5. Expanding Editing Access Across Elite Tomato Genotypes

The breeding value of genome editing depends on its application to elite and locally adapted tomato genotypes, but transformation and regeneration remain strongly genotype-dependent. Expression of the developmental regulators *SlGRF4* and *SlGIF1* improved callus transformation and shoot regeneration in three tested genotypes, although the magnitude of the response varied among them [58]. A transient in planta approach generated chimeric T_0_ mutants at 11.7% frequency without conventional stable transformation and tissue-culture regeneration, but researchers did not establish routine recovery of uniform, germline-transmitted alleles [59]. Future systems should demonstrate reproducible editing across diverse cultivars, stable germline transmission, recovery of non-chimeric progeny, and reliable exclusion of editing-vector integration. Regeneration-associated variation should also be assessed using appropriate regenerated controls.

### 7.6. Establishing Evidence Standards for Trade-Off Resolution

Future studies should distinguish technical, biological, and breeding success. Technical success requires recovery and complete molecular characterization of the intended allele; biological success requires demonstrating the predicted molecular and physiological response; and breeding success requires improving the target trait without unacceptable penalties across relevant genetic backgrounds, environments, generations, and production systems. Evidence should include independently generated edited events, appropriate segregant or regeneration-matched controls, complete target-locus characterization, stable inheritance, and concurrent measurement of the intended and biologically plausible competing traits. Claims of “no yield penalty” or “no quality penalty” require adequate replication and statistical power and, where appropriate, predefined equivalence or non-inferiority criteria. Trade-off mitigation should therefore be demonstrated as a stable multi-trait outcome rather than inferred from a single edited line or environment.

## 8. Conclusions

Genome editing provides unprecedented control over defined tomato genes and alleles, but molecular precision does not automatically produce a balanced breeding outcome. Tomato yield, fruit quality, reproductive development, plant architecture, stress adaptation, and defence are connected through pleiotropic genes, redundant paralogues, dosage-sensitive regulatory networks, assimilate allocation, linkage, epistasis, and genotype-by-environment interactions. Consequently, an edit that improves one characteristic may impose a cost on another trait, developmental stage, genetic background, or production environment.

This review shows that the editing strategy should match the trade-off’s causal architecture. Complete loss-of-function editing is most appropriate when strong suppression of a nonessential negative regulator or susceptibility factor is required. Multiplex editing may be needed to overcome functional redundancy, but single, intermediate, and complete mutant combinations should be compared to distinguish additive effects from epistasis. For pleiotropic or dosage-sensitive genes, cis-regulatory editing, partial-function alleles, or tissue-, stage-, or environment-responsive regulation may preserve essential functions while moderating undesirable effects. Base editing, prime editing, and direct allele repair are particularly valuable when a favourable phenotype depends on a validated causal nucleotide or defined sequence change.

Available tomato studies demonstrate that genome editing can weaken some difficult breeding relationships, including associations between sweetness and fruit size or yield. Nevertheless, claims of trade-off resolution must remain restricted to the traits and conditions evaluated. A nonsignificant difference in a limited experiment does not establish biological equivalence or the absence of hidden costs. Validation should connect the intended edit with molecular function and measure relevant competing phenotypes, including flowering, fruit set, fruit number, individual fruit weight, total and marketable yield, seed production, fruit composition, sensory quality, postharvest behaviour, and stress performance. Independent edited events, suitable comparators, stable inheritance, and evaluation across relevant genotypes and environments are essential for distinguishing technical editing success from breeding improvement.

Breeding deployment also requires recipient-genotype sequence information, reliable transformation or delivery, recovery of genetically uniform and heritable plants, and confirmation that the final product retains acceptable performance after segregation, crossing, or trait stacking. Structural variation, cultivar-specific haplotypes, regeneration responses, and regulatory requirements should therefore be considered during target selection rather than after producing an edited phenotype.

In conclusion, the value of genome editing in tomato breeding lies in quantitative biological optimization rather than indiscriminate gene disruption. A trade-off-aware framework linking causal-allele identification, mechanism-matched editing, multidimensional phenotyping, and breeding-level validation provides a rigorous basis for developing tomato cultivars that balance productivity, fruit quality, resilience, reproductive performance, and commercial suitability. An edited allele should be considered to have mitigated a trade-off only when its intended benefit remains stable without an unacceptable redistribution of biological cost to another trait or environment.

## Figures and Tables

**Table 1:** Additional representative genome-editing outcomes and remaining trade-offs in tomato.

Trait Balance	Target and Editing Strategy	Reported Outcome	Remaining Limitation	References
Compact growth versus genetic BACKGROUND dependence	Base editing to repair *SSP2*	Produced compact plants and increased early fruit yield	Transferability across diverse breeding backgrounds remains to be tested	[13]
Powdery mildew resistance versus productivity	*SlMlo1* knockout	Conferred powdery mildew resistance; plant morphology and harvested fruit weight were comparable to the wild type under the tested conditions.	Evidence is limited to the tested genotype and experimental conditions.	[23]
Broad-spectrum disease resistance versus growth	*SlDMR6-1* knockout	Increased resistance to multiple bacterial, oomycete, and fungal pathogens	Agronomic performance of uninfected edited plants has not been established under field conditions.	[32]
Heat-resilient fruit set versus seed production	*SlAGL6* knockout	Enabled facultative parthenocarpic fruit development when heat stress impaired fertilization	Fruits formed without fertilization were seedless; seed production remained possible when fertilization succeeded	[33]

**Table 2:** Core evidence required to validate mitigation of trait trade-offs in genome-edited tomato.

Validation Domain	Evidence Required	Essential Comparison	References
Intended and competing traits	Target-trait improvement together with relevant effects on yield, reproduction, fruit quality, growth, and stress responses	Edited lines compared with the unedited recipient and an appropriate breeding comparator	[12,31]
Edit identity and inheritance	Intended sequence, zygosity, local sequence integrity, construct absence where claimed, and stable transmission	Independent edited events and their progeny compared with the unedited recipient	[23,50]
Tissue and developmental resolution	Molecular and phenotypic effects measured in the relevant tissue and developmental stage	Tissue- and stage-matched samples, with comparisons to whole-organ or bulk measurements where appropriate	[44,45]
Genetic background and environmental stability	Reproducibility of the intended and competing phenotypes across cultivars, environments, and generations	Replicated evaluation in breeding-relevant backgrounds under control and production- relevant conditions	[49,51]

**Table 3:** Deployment and regulatory requirements for genome-edited tomato lines.

Category	Assessment Basis	Key Limitation	References
Breeding transfer or reconstruction	Identity of the edited allele, recovery of the recipient genetic background, retention of useful traits, and stable agronomic performance	Edited wild or experimental accessions are not automatically breeding-ready commercial cultivars	[7,24]
Final-product characterization	Complete sequence and allelic state, identity of each component edit, segregation of editing constructs, trait-specific phenotypic and compositional evidence, and seed identity and purity	DNA-free or transgene-free status alone does not establish agronomic value or regulatory status	[14,23]
Post-release evidence and communication	Allele and seed identity, trait stability, agronomic performance, and evidence supporting product claims across seed lots, production regions, seasons, and generations	Regulatory eligibility or market introduction does not independently establish agronomic superiority, health benefits, or general consumer acceptance	[53,56]
European Union classification	Final-product sequence, modification type and number, equivalence criteria, and trait eligibility determine category 1 verification or category 2 requirements	Regulation (EU) 2026/1388 applies generally from 17 July 2028; until then, the existing GMO framework applies. Absence of foreign DNA alone does not establish category 1 status	[54]
Japanese notification	Eligible genome-edited foods are assessed and notified on a product- and line-specific basis; high-GABA tomato lines 87-17 and 206-4 are listed separately	Notification of one edited line does not determine the status of another line and should not be described as conventional GMO approval	[55]

## Data Availability

No new data were created or analyzed in this study. Data sharing is not applicable to this article.
